# Supplementary material for: Molecular and clinicopathological differences between depressed and protruded T2 colorectal cancer
Source: PLoS One. 2022 Oct 20;17(10):e0273566. doi: 10.1371/journal.pone.0273566 (PMC9584453; doi:10.1371/journal.pone.0273566)
Supplement: Attachment — Submitted filename: Response to Reviewers.docx [file pone.0273566.s001.docx]

*PLOS ONE*

Dear Editors,

We thank the reviewers for their careful assessment of our manuscript and their useful comments. In response to the Reviewers’ comments, we have carefully revised the manuscript point-by-point.

We hope that our manuscript is now suitable for publication in the *International Journal of PLOS ONE* and look forward to hearing from you at your earliest convenience.

Yours sincerely,

Our responses to the Reviewers’ reports are as follows:

----------------------------------------------------------------------

Response to the Reviewer

----------------------------------------------------------------------

Thank you for your careful review of our paper. We have responded to each of your points below.

Reviewers' comments:

Reviewer #1:

Major comments:

Mochizuki et al. analyzed 30 depressed types and 25 protruded type T2 colorectal cancers by immunohistochemistry-based CMS classification. CMS4 tumors were exclusive to the depressed type, and malignant pathological findings were more commonly observed in depressed type tumors compared with protruded type tumors.
I do not understand why the authors excluded flat type and ulcerative type lesions from the analysis. The comparison between depressed and protruded types is insufficient to determine the clinicopathological features of depressed type lesions among T2 colorectal cancers. CMS4 and malignant pathological findings may be significantly less common in protruded type lesions among T2 tumors. Thus, the authors’ conclusion that depressed-type T2 colorectal cancers are associated considerably with CMS4 is not justified by the presented data.

Response: Thank you for your helpful comment. This study excluded flat and ulcerative types and focused our analysis on protruded and depressed types. The adenoma-carcinoma sequence and the de novo pathway, which are the representative carcinogenic pathway of CRC, have been reported to have protruded and depressed morphologies, respectively. To investigate the molecular biological characteristics of these two types, the present analysis focused on the protruded and depressed types.[1]. Attached are figures (Fig. 1) on two representative CRC carcinogenesis pathways reported by Kudo et al. As you pointed out, there is a hidden possibility that CMS4 and malignant pathological findings are less common in the protruded type. However, we believe that all CMS4 colorectal cancers were depressed-type colorectal cancers is very impactful data. We want to add flat or ulcerative lesions to our analysis as future work. We have made changes to the Introduction based on your suggestions. We have changed the description of the adoption of morphology as follows:

“The adenoma–carcinoma sequence and the de novo pathway, which are representative carcinogenic pathways of CRC, are reportedly associated with the protruded and depressed morphologies, respectively (Fig. 1). The present analysis focused on the molecular characteristics of the protruded and depressed types[14].” (Page 5, line 83)

Fig. 1 ‘‘Adenoma-carcinoma sequence’’ and ‘‘de novo’’ theory.

Minor comments:

Protein symbols should not be italicized.

Response: Thank you for your comment. We corrected all italicization of proteins.

Reviewer #2:

Major comments:

1. In this study, the authors focused on just depressed and protruded types of the four gross morphological classifications of T2 CRC. Why did the authors select these types? Also, why these 2 types were distinguished by molecular profiling? Please discuss and address about it in Introduction or Discussion section from clinical or molecular point of view.

Response: Thank you for your useful comment. In this study, our primary objective was to elucidate the molecular biological characteristics of depressed type colorectal cancer, which is reported to have a high malignant potential, and we referred to the research methods of the pioneering Kudo et al. The adenoma-carcinoma sequence and the de novo pathway, which are representative carcinogenic pathway of CRC, have been reported to have protruded and depressed morphologies, respectively. In order to investigate the molecular biological characteristics of these two types, the present analysis focused on the protruded and depressed types[1]. As you pointed out, there is a hidden possibility that CMS4 and malignant pathological findings are less common in the protruded type. However, we believe that all CMS4 colorectal cancers were depressed-type colorectal cancers is very impactful data. We want to add flat or ulcerative lesions to our analysis as future work. We have made changes to the Introduction based on your suggestions. We have changed the description of the adoption of morphology as follows:

“The adenoma–carcinoma sequence and the de novo pathway, which are representative carcinogenic pathways of CRC, are reportedly associated with the protruded and depressed morphologies, respectively (Fig. 1). The present analysis focused on the molecular characteristics of the protruded and depressed types [14].” (Page 5, line 83)

Fig. 1 ‘‘Adenoma-carcinoma sequence’’ and ‘‘de novo’’ theory.

We also believe that molecular profiling has demarcated these two types as follows. First, Kudo et al. reported that depressed CRC is characterized by higher expression of genes related to angiogenesis and EMT. This is consistent with the characteristics of CMS4, and in this study, all of the patients classified as CMS4 were depressed type. Based on these findings, we hypothesized that depressed type CRC is more strongly associated with CMS4 compared to the protruded type. We have added the description of the molecular profiling of morphology as follows:

“The concordance of the molecular characteristics of CMS4 with those of depressed CRC, and the classification of all CMS4 cases as the depressed type, demonstrated the strong association between CMS4 and depressed CRC.” (Page 15, line 275)

2. The authors showed the relationship between expression of CDX2, KER and malignant grade of CRC. It is likely that both genes are associated with EMT pathway. Please discuss about molecular mechanism in detail. Also, please cite some papers regarding the association between both genes and EMT.

Response: Thank you for your very important point. Yu et al. reported that CDX2 inhibits EMT and metastasis of CRC by regulation of Snail expression and β-catenin stabilization through PI3K/Akt/GSK-3β signaling. Kim et al. also reported that keratin loss due to phosphorylation is associated with EMT. Based on the above, we have added the description of CDX2 and EMT as follows:

“It has also been reported that CDX2 expression is inversely correlated with the malignancy of CRC [19] and inhibits EMT and metastasis[20, 21] by regulating Snail expression and β-catenin stabilization via PI3K/Akt/GSK-3β signaling [22].” (Page 14, line 259)

We have added two references reporting on the association between CDX2 and EMT to page 14, line 261, as follows:

20.	Liu H, Yan R, Liang L, Zhang H, Xiang J, Liu L, et al. The role of CDX2 in renal tubular lesions during diabetic kidney disease. Aging (Albany NY). 2021; 13(5): 6782-6803. https://doi.org/10.18632/aging.202537. PMID: 33621200.

21.	Zhang JF, Qu LS, Qian XF, Xia BL, Mao ZB, Chen WC. Nuclear transcription factor CDX2 inhibits gastric cancercell growth and reverses epithelialtomesenchymal transition in vitro and in vivo. Mol Med Rep. 2015; 12(4): 5231-5238. https://doi.org/10.3892/mmr.2015.4114. PMID: 26238762.

On the other hand, Kim et al. reported that keratin loss due to phosphorylation is associated with EMT. Based on this, we have added the description of KER and EMT and a reference as follows:

“On this basis, it has been reported that keratin loss due to phosphorylation is associated with EMT [24].” (Page 15, line 267)

24.	Kim HJ, Choi WJ, Lee CH. Phosphorylation and Reorganization of Keratin Networks: Implications for Carcinogenesis and Epithelial-Mesenchymal Transition. Biomol Ther (Seoul). 2015; 23(4): 301-312. https://doi.org/10.4062/biomolther.2015.032. PMID: 26157545

3. The authors showed no relationship between each CMS's tumor location (rectum or not). How about is sidedness of colon cancer? Recently, the impact of sidedness of CRC on tumor immunity has been reported.

Response: Thank you for your critical comment. We did an additional analysis of the right or left side of the colon and whether it was rectal or not. The results were as follows, with no significant differences. This may be due to the small sample size. Since the relationship between tumor location and immunity is an essential point, we would like to study it again in the future with a more significant number of cases.

|  | Depressed  (n = 30) | Protruded  (n = 25) | Total  (n = 55) | *P*-value |
| --- | --- | --- | --- | --- |
| Location (right sided) | 5 (16.6%) | 9 (36.0%) | 14 (25.5%) | 0.128 |

|  | CMS1  (n = 3) | CMS2/3  (n = 37) | CMS4  (n = 15) | *P*-value |
| --- | --- | --- | --- | --- |
| Morphology (depressed) | 0 (0.0%) | 15 (40.5%) | 15 (100%) | < 0.001 |
| Location (right sided) | 2 (66.7%) | 10 (27.0 %) | 2 (13.3%) | 0.143 |

4. The authors described depressed CRC as malignant behavior requiring careful surveillance. How do the authors plan to use findings obtained from this study in clinical practice? For example, can depressed-type T2 CRC with CMS4 be a candidate factor for the indication for adjuvant chemotherapy? Please describe the clinical perspective of this study.

Response: Thank you for your helpful comment. CMS4 has a poor prognosis and is also often at an advanced stage (III-IV)[2]. Considering the high pathologic grade of the depressed type, depressed CRC with CMS4 is likely to require chemotherapy. Based on the results of this study alone, it is not possible to discuss chemotherapy and its effect on chemotherapy due to the small number of cases. In the future, we would like to collect prospective cases and study depressed colorectal cancer, CMS4, and the effects of chemotherapy. We have added the description of a future challenge as follows:

“CMS4 is associated with a poor prognosis and is often not detected until the advanced stages (III-IV)[4]. Considering the high pathological grade of the depressed type, depressed CRC with CMS4 usually requires chemotherapy. Although the association between the CMS and long-term outcomes could not be demonstrated due to the small number of cases in this study, we plan to accumulate more cases prospectively as a practical application of this study to determine whether morphology and CMS classification are candidate predictors of the need for adjuvant chemotherapy.” (Page 16, line 293)

Minor comments:

1. In the introduction section, the authors described the epidemiology of CRC. More worldwide information is needed, not just focusing on Japanese data.

Response: Thank you for your helpful comment. We have added the description of the epidemiology of CRC as follows:

“It is the third most commonly diagnosed malignancy in men and the second in women, with more than 1.8 million new cases and 880,000 deaths reported in 2018 [1].” (Page4, line 51)

2. We can find some typographical errors and different font size (e.g line 227 and 300). Please correct them.
Response: Thank you for pointing this out. We have corrected the font.

3. Who determined the morphological type? Some experts of endoscopists probably did; please specify it in the method section because it sometimes depends on the endoscopist.

Response: Thank you for your helpful comment. We have added the description of the method of determining the morphological type as follows:

“The morphological type was retrospectively determined by several endoscopic experts (K.M., K.K., Y.O., Y.K., H.M.).” (Page6, line 111)

4. We miss the data about long-term outcomes in this study. The reviewers think it is a weakness of this study. Please address it in the Discussion section.

Response: Thank you for your helpful comment. Long-term outcomes were analyzed, but the results did not differ due to the small number of cases. Figure B and C of the analysis results are attached. We have added the description of the discussion as follows:

“Although the association between the CMS and long-term outcomes could not be demonstrated due to the small number of cases in this study, we plan to accumulate more cases prospectively as a practical application of this study to determine whether morphology and CMS classification are candidate predictors of the need for adjuvant chemotherapy.” (Page16, line 295)

Figure B; Relationship between CMS and overall survival

P =0.4, Compared to the Log-lank test

*dep, depressed; prot, protruded

Figure C; Relationship between CMS and disease-free survival

P =0.7, Compared to the Log-lank test

*dep, depressed; prot, protruded

**1.**	Kudo SE, Takemura O, Ohtsuka K. Flat and depressed types of early colorectal cancers: from East to West. Gastrointest Endosc Clin N Am. 2008; 18(3): 581-593, xi. <https://doi.org/10.1016/j.giec.2008.05.013>. PMID: 18674705.

**2.**	Guinney J, Dienstmann R, Wang X, de Reynies A, Schlicker A, Soneson C, et al. The consensus molecular subtypes of colorectal cancer. Nat Med. 2015; 21(11): 1350-1356. <https://doi.org/10.1038/nm.3967>. PMID: 26457759.
