# Supplementary material for: Molecular and clinicopathological differences between depressed and protruded T2 colorectal cancer
Source: PLoS One. 2022 Oct 20;17(10):e0273566. doi: 10.1371/journal.pone.0273566 (PMC9584453; doi:10.1371/journal.pone.0273566)
Supplement: Attachment — Submitted filename: Response to Reviewers.docx [file pone.0273566.s002.docx]

*PLOS ONE*

Dear Editors,

We thank the reviewers for carefully assessing our manuscript and their valuable comments. In response to the Reviewer’s comments, we have carefully revised the manuscript point-by-point.

We hope that our manuscript is now suitable for publication in the *International Journal of PLOS ONE* and look forward to hearing from you at your earliest convenience.

Yours sincerely,

Our responses to the Reviewer’s reports are as follows:

----------------------------------------------------------------------

Response to the Reviewer

----------------------------------------------------------------------

Thank you for your careful review of our paper. We have responded to each of your points below.

**Reviewer's comments:**

**Reviewer #1:**

This study analyzes immunohistochemistry-based consensus molecular subtype classification in depressed- and protruded-type T2 colorectal cancers and indicate that depressed-type T2 CRCs are more likely to show the CMS4 phenotype and malignant pathological findings.

If the authors want to elucidate the “molecular and clinicopathological features of depressed T2 colorectal cancer”, they should also analyze flat- and ulcerative-type lesions. Otherwise, the authors should extensively revise the manuscript, including the title, to clearly indicate that this study analyzed the differences between depressed- and protruded-type T2 colorectal cancer.

The authors added Figure 1, suggesting that protruded and depressed lesions are related to two major tumorigenic pathways. However, what are the tumorigenic processes of flat and ulcerative lesions, which constituted 49 cases in this cohort?

**Response :** Thank you for your perceptive comment. We changed the title to “Molecular and clinicopathological differences between depressed and protruded T2 colorectal cancer.” As you have concerned in the original manuscript, we have excluded findings related to the flat and ulcerated types, which should be discussed as the major limitation in this paper. Therefore, we have added a paragraph to discuss it as follows:

“Fourth, we excluded flat type and ulcerative type cases of CRC. In this study, we focused on the two hypotheses of carcinogenic pathways in CRC: protruded type by the adenoma-carcinoma sequence and depressed type by the de novo pathway. We speculate that lesions with endoscopically depressed localization rarely have an adenoma component in the pathology[11] and take their carcinogenic pathway. The flat type and ulcerative type are comprised of a mixture of both protruded and depressing elements and a mixture of tumors that have followed many carcinogenic pathways, such as those originating from serrated lesions[30] or granular-type laterally spreading tumors (G-LST)[31]. Therefore, it was appropriate first to clarify the two representative carcinogenic pathways of CRC, the adenoma-carcinoma sequence and the de novo pathway, and clarification of the flat and ulcerative type was considered an issue to be addressed later. We excluded flat and ulcerated type T2 CRCs. However, there were 49 cases of flat and ulcerated type, which is not negligible and may have a large selection bias. Serrated pathway carcinogenesis has been reported to include MSI carcinomas[30], and G-LST has been reported to have a High prevalence of CpG island methylator phenotype-high[31]. These are consistent with the characteristics of CMS1. Perhaps the morphology excluded in this study includes CMS1 cases, which may have represented only a minority of cases in this study.” (Page 16, line 319)

11.	Kudo SE, Kouyama Y, Ogawa Y, Ichimasa K, Hamada T, Kato K, et al. Depressed Colorectal Cancer: A New Paradigm in Early Colorectal Cancer. Clin Transl Gastroenterol. 2020; 11(12): e00269. https://doi.org/10.14309/ctg.0000000000000269. PMID: 33512809.

30.	O'Brien MJ, Zhao Q, Yang S. Colorectal serrated pathway cancers and precursors. Histopathology. 2015; 66(1): 49-65. https://doi.org/10.1111/his.12564. PMID: 25263173.

31.	Hiraoka S, Kato J, Tatsukawa M, Harada K, Fujita H, Morikawa T, et al. Laterally spreading type of colorectal adenoma exhibits a unique methylation phenotype and K-ras mutations. Gastroenterology. 2006; 131(2): 379-389. https://doi.org/10.1053/j.gastro.2006.04.027. PMID: 16890591.
